# A donor splice site mutation in *CISD2* generates multiple truncated, non-functional isoforms in Wolfram syndrome type 2 patients

**DOI:** 10.1186/s12881-017-0508-2

**Published:** 2017-12-13

**Authors:** Monica Cattaneo, Lucia La Sala, Maurizio Rondinelli, Edoardo Errichiello, Orsetta Zuffardi, Annibale Alessandro Puca, Stefano Genovese, Antonio Ceriello

**Affiliations:** 1 Cardiovascular Research Unit, IRCCS MultiMedica, Via G. Fantoli 16/15, 20138 Milan, Italy; 2 Diabetes Endocrine and Metabolic Diseases Unit, IRCCS MultiMedica, 20099 Sesto San Giovanni, Milan, Italy; 3 Department of Molecular Medicine, University of Pavia, 27100 Pavia, Italy; 4 Department of Medicine and Surgery, University of Salerno, 84084 Salerno, Italy; 5 Institut d’Investigacions Biomèdiques August Pi i Sunyer (IDIBAPS) and Centro de Investigación Biomedica en Red de Diabetes y Enfermedades Metabólicas Asociadas (CIBERDEM), Barcelona, Spain; 6 IRCCS Centro Cardiologico Monzino Diabetes, Endocrine and Metabolic Diseases Unit, 20138 Milan, Italy

**Keywords:** *CISD2*, Wolfram syndrome type 2, mRNA splicing, Non functional isoforms, Nonsense-mediated mRNA decay

## Abstract

**Background:**

Mutations in the gene that encodes CDGSH iron sulfur domain 2 (*CISD2*) are causative of Wolfram syndrome type 2 (WFS2), a rare autosomal recessive neurodegenerative disorder mainly characterized by diabetes mellitus, optic atrophy, peptic ulcer bleeding and defective platelet aggregation. Four mutations in the *CISD2* gene have been reported. Among these mutations, the homozygous c.103 + 1G > A substitution was identified in the donor splice site of intron 1 in two Italian sisters and was predicted to cause a exon 1 to be skipped.

**Methods:**

Here, we employed molecular assays to characterize the c.103 + 1G > A mutation using the patient’s peripheral blood mononuclear cells (PBMCs). 5′-RACE coupled with RT-PCR were used to analyse the effect of the c.103 + 1G > A mutation on mRNA splicing. Western blot analysis was used to analyse the consequences of the CISD2 mutation on the encoded protein.

**Results:**

We demonstrated that the c.103 + 1G > A mutation functionally impaired mRNA splicing, producing multiple splice variants characterized by the whole or partial absence of exon 1, which introduced amino acid changes and a premature stop. The affected mRNAs resulted in either predicted targets for nonsense mRNA decay (NMD) or non-functional isoforms.

**Conclusions:**

We concluded that the c.103 + 1G > A mutation resulted in the loss of functional CISD2 protein in the two Italian WFS2 patients.

**Electronic supplementary material:**

The online version of this article (10.1186/s12881-017-0508-2) contains supplementary material, which is available to authorized users.

## Background

Wolfram syndrome type 2 (WFS2 OMIM #604928) is a rare autosomal recessive neurodegenerative disorder that affects the function of several organs as well as, neurons and pancreatic beta cells. It is characterized by diabetes mellitus, optic atrophy, deafness peptic ulcer bleeding and defective platelet aggregation [1–3]. The affected gene in WFS2 is *CISD2,* an evolutionarily conserved gene that is localized to chromosome 4q24 close to a genetic component implicated in human longevity [4]. The gene consists of three small exons distributed over 23.82 Kb of genomic DNA, generating a transcript of 2327 nucleotides characterized by a long 3′-UTR tail (NM_001008388.4; ENST00000273986). Beyond the principal transcript, two other poorly supported splice variants have been annotated (ENST00000503643, ENST00000574446). These variants diverge from the canonical mRNA in the N-terminal region and the 3′-UTR tail.

The principal transcript encodes an iron sulfur (Fe-S) protein that consists of an N-terminal transmembrane helix and a C-terminal CDGSH domain containing the Fe-S cluster [5]*.* It displays a dynamic subcellular localization between the endoplasmic reticulum (ER) and mitochondrial membranes, and is specifically enriched in the mitochondrial outer membrane (MOM) and the mitochondria-associated ER membranes (MAMs) [6]. The exact function of the CISD2 protein is still unknown but it is reported to be an iron donating protein, that can transfer iron to the mitochondria in living cells, and to have a crucial role in the regulation of iron and reactive oxygen species (ROS) as well as in preserving and maintaining mitochondrial homeostasis [7]. Indeed, the downmodulation of CISD2 in several cell lines resulted in decreased mitochondrial function and integrity as well as the activation of autophagy and apoptosis [8–10]. The mouse *Cisd2* knockout, which is an available disease model that recapitulates several clinical manifestations of WFS2, further emphasized the association of CISD2 function with mitochondrial integrity and homeostasis, establishing WFS2 as a mitochondrial-mediated disorder [11]. Mitochondrial breakdown and dysfunction are reported to be the early promoters of premature ageing and autophagic cell death in deficient mice [11].

To date, four cases of WFS2 with mutations in *CISD2* have been reported: *i.* a homozygous missense mutation identified in three consanguineous families of Jordanian descent, located close to the intron-exon junction of exon 2, that affects mRNA splicing and causes a truncated non-functional protein [1]; *ii.* a homozygous intragenic deletion affecting the entirety of exon 2 in a European Caucasian girl [2]; *iii.* a homozygous mutation (NM_001008388.4:c.103 + 1G > A) in the donor splice site of intron 1 that was predicted to cause exon 1 to be skipped identified in two Italian siblings [3]; and *iv.* a homozygous missense variant located in exon 2 that is predicted to induce a conformational change of the CISD2 homodimeric protein model and to alter the redox and functional properties of the protein in a Moroccan patient [12].

Here, we report the molecular and biochemical characterization of the c.103 + 1G > A mutation using peripheral blood mononuclear cells (PMBCs) isolated from the affected siblings and unaffected parents. The results provide mechanistic insight into the effect of the mutation on the *CISD2* splicing pattern and on the mRNA and protein levels.

## Methods

### Peripheral blood mononuclear blood cells isolation

PBMCs were isolated from the whole venous blood of the healthy donator, patients and parents by density gradient centrifugation using Histopaque (Sigma-Aldrich, Milan, Italy), according to the manufacturer’s instructions. Written informed consent was obtained from all subjects enrolled in the study.

### RNA extraction, RT-PCR and quantitative real-time PCR

Total RNA was extracted using miRNeasy mini Kit (Qiagen, Milan Italy) and reverse transcribed with Superscript III reverse transcriptase (Invitrogen, Milan, Italy), according to manufacturer’s instructions.

Semi-quantitative PCR amplifications were performed using GoTaq DNA Polymerase (Promega, Milan, Italy). The PCR conditions were: denaturation at 95 °C for 2 min, followed by 24 (for *actin B*, *ActB*) and 35 (for *CISD2*) cycles at 95 °C for 30 s, 60 °C for 40 s, and 72 °C for 60 s.

Quantitative real-time PCR was performed in triplicate using ABI 7900 HT thermo cycler and SYBR Green PCR Master mix (Applied Biosystems, Life Technology, Monza, Italy). Data were normalized to *ActB* expression using the delta Ct method.

Primer Sequences:

- ex1-F: 5′-gtccctgaaagcattaccgg-3′
- ex2-F: 5′-cagaatggcttcggttattg-3′
- ex2int-F: 5′-tcctcccgaagaagaaacaaca −3′
- ex2–3-F: 5′-gttctaaaacgtttcctgcc-3′
- 3′-utr-R: 5′-cattccaccccagctgtcac-3′
- Ex3-R: 5′- tatgtgaaccatcgcaggca-3′
- ActB-F: 5′-cagccatgtacgttgctatccagg-3
- ActB-R: 5′-aggtccagacgcaggatggcatg −3′

### Rapid amplification of 5′ cDNA ends (5′-RACE)

The SMARTer RACE cDNA Amplification kit (Clontech, California, USA) was used to extend the *CISD2* mRNA in the PBMCs from the patients, according to the manufacturer’s instructions. The synthesis of the first strand cDNA was carried out with total RNA (1 μg) using a 5′ CDS primer A, SMART II A oligonucleotide and SMARTScribe Reverse Transcriptase. The 5′-RACE PCR reaction was performed with the high fidelity enzyme (SeqAmp DNA polymerase, Clontech) using a universal primer A mix (UPM, forward primer) and an antisense gene specific primer (GSP1) designed against the exon 3 of the *CISD2.* The nested 5′-RACE PCR was carried out using a specific reverse primers (GSP2) designed inner to the exon 3 of *CISD2*. The primer sequences are noted below (the bold sequences referred to the 15 nucleotides overlapped withpUC19 vector suitable for the in fusion cloning). The 5′-RACE products were separated by 2% agarose gel electrophoresis and the resulting bands were extracted from the gel, purified using NucleoSpin gel-PCR Clean Up Kit (Clontech), and cloned into pUC19. The positive clones that contained the inserts of the expected size were screened by colony PCR using M13F and M13R primers and sequenced by the Sanger method (BigDye Terminator v 3.1) and 3730XL DNA Analyzer (Applied Biosystems).

Primer Sequences:

- GSP1: 5′-**gattacgccaagctt**tcttcttcagtattagtggacccac-3′
- GSP2: 5′-**gattacgccaagctt**tgtgaaccatcgcaggcaggaaacg-3′
- M13-F: 5′-gtaaaacgacggccagt-3′
- M13-R: 5′-caggaaacagctatgac-3′

### SNP genotyping

The genomic DNA was isolated from buccal swab using QiAamp DNA Mini Kit (Qiagen, Milan, Italy). The genomic region flanking SNP was amplified with the high fidelity enzyme (SeqAmp DNA polymerase, Clontech) using primers noted below. The PCR products were sequenced by the Sanger method (BigDye Terminator v 3.1) and 3730XL DNA Analyzer (Applied Biosystems).

Primer sequences:

- SNP-F: 5′-gcagtccgccgcgagcgtac-3′
- SNP-R: 5′-ccggtaatgctttcagggac-3′

### Western blot

PBMCs were lysed in 50 mM Tris HCl (PH 7.6), 150 mM NaCl, 1% Nonidet P-40 containing protease and phosphatase inhibitor cocktail (Sigma). The protein concentration was determined by Bradford assay (Thermo Scientific, Milan, Italy). The total proteins were resolved on 14% SDS-Polyacrylamide gel, blotted onto PVDF membrane (GE Healthcare, Euroclone, Pero, Milan, Italy), and probed with the antibodies anti-CISD2 (1:1000, Proteintech, LaboSpace Srl, Milan, Italy) and β-tubulin (1:2000, Sigma). Proteins were detected with anti-rabbit secondary antibodies (1:100,000, GE Healthcare) and anti-mouse (1:10,000, GE Healthcare). Filters were developed with western Sure premium Chemiluminescent Substrate LI-COR using LI-COR instrument. β tubulin was used as normalizer.

### Statistical analyses

Statistically significant differences between samples were determined in all the experiments by one-way ANOVA analyses; *, **, *** and **** indicated *p* < 0.05, *p* < 0.01 and *p* < 0.001, *p* < 0,0001, respectively. A *p* value < 0.05 was considered statistically significant.

## Results

### Molecular analysis of *CISD2* mRNA in PBMCs

**Fig. 1:**
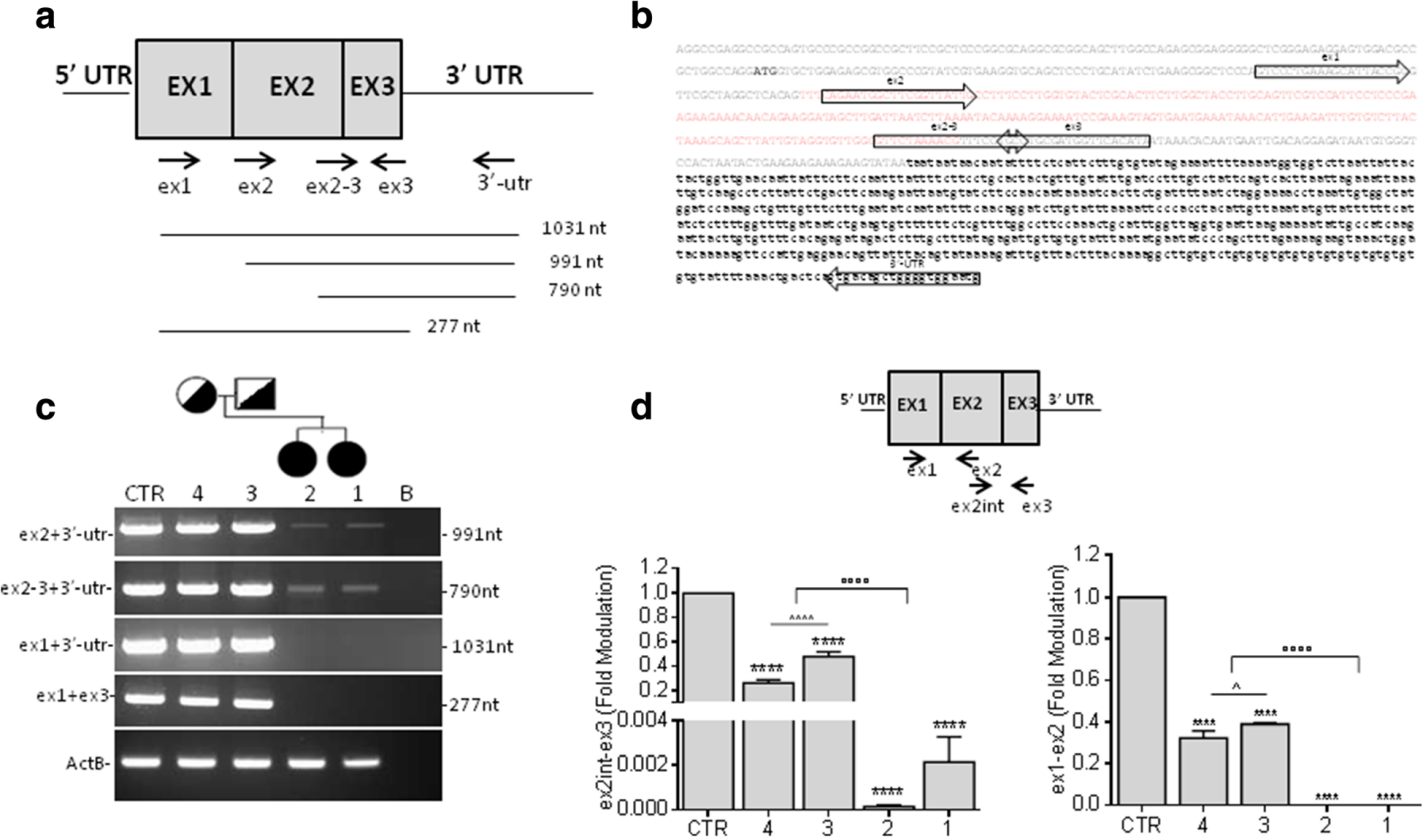
Molecular characterization of the homozygous and heterozygous *CISD2* transcript. **a** A schematic representation of the *CISD2* transcript: the arrows represent the PCR primers, and four overlapping sets of *CISD2* primers were used. The lines indicate the expected sizes of the PCR products using the specific set of primers. **b** The nucleotide sequence of *CISD2* cDNA with exons in uppercase letters and alternate colours, the 5′-UTR and 3′-UTR in lowercase letters, and the ATG in bold letters. The *arrows* indicate the PCR primers used for the characterization of the *CISD2* transcript. **c** RT-PCR analysis performed on PBMCs derived from a healthy individual (CTR), homozygous probands (1 and 2) and heterozygous unaffected parents (3 and 4). *ActB* was used as a loading control, and a negative PCR control (**b**) was included to test possible contamination. The data indicate a putative skipping of the N-terminus of *CISD2* in patients and suggest a strong instability of mutant *CISD2* mRNA. **d** mRNA quantitative real-time PCR. A schematic representation of the *CISD2* transcript shows the PCR primers used for the analysis. The *CISD2* mRNA level drastically decreased in the homozygous samples, compared to that in the healthy PBMCs. The histograms show values normalized relative to a housekeeping gene (*ActB*) and expressed as a fold modulation compared to the healthy control. The error bars represent the mean ± SEM for three experiments. Ordinary one-way ANOVA, *P < 0,05, **P < 0,01, ***P < 0,001 and ****P < 0,0001

It was previously predicted that a homozygous *CISD2* mutation in the donor splice site of intron 1 (NM_001008388.4:c.103 + 1G > A) caused exon 1 to be skipped in two Italian sisters affected by WFS2 [3]. To analyse the effect of the c.103 + 1G > A mutation on the mRNA, a reverse transcription polymerase chain reaction (RT-PCR) assay covering almost the entire cDNA was performed on RNA extracted from PMBCs derived from the patients (1 and 2) and the heterozygous unaffected parents (3 and 4), respectively. The PBMCs derived from healthy donor were also included as a control (CTR). Overlapping primers designed against each of the three exons of the *CISD2* gene were coupled with two primers designed against the 3′-UTR and exon 3, allowing for the dissection of four fragments representing progressive 5′ deletions (Fig. 1a and b). The primers designed against exons 2 and 3 amplified the expected PCR products of 991 and 790 nt in all samples, although very faint levels were detected in the patients (Fig. 1c). As predicted, no amplification was observed in patients using the primers to amplify the *CISD2* region from exon 1 to exon 3, while PCR products of the expected size (1031 and 227 nt) were obtained in the remaining samples (Fig. 1c). The quantitative PCR analysis performed with the primer set designed against exons 2 and 3, confirmed the reduced amounts of *CISD2* mRNA amplified in the patients compared with those obtained in either the parents or the healthy control (less than 99%) (Fig. 1d), suggesting the strong instability of the mutant mRNA. A decrease in mRNA levels of approximately 64% was also observed in the heterozygous samples compared to the mRNA levels of the control (Fig. 1d). Finally, the primer set designed to amplify exon 1 validated a decrease of *CISD2* mRNA of approximately 65% in the parents and undetectable signals in the homozygous samples (Fig. 1d).

Together, the results indicate a potential skipping of exon 1 in the transcripts of the patients, which severely affects the mRNA stability.

### Identification of multiple *CISD2* isoforms by 5′-RACE

**Fig. 2:**
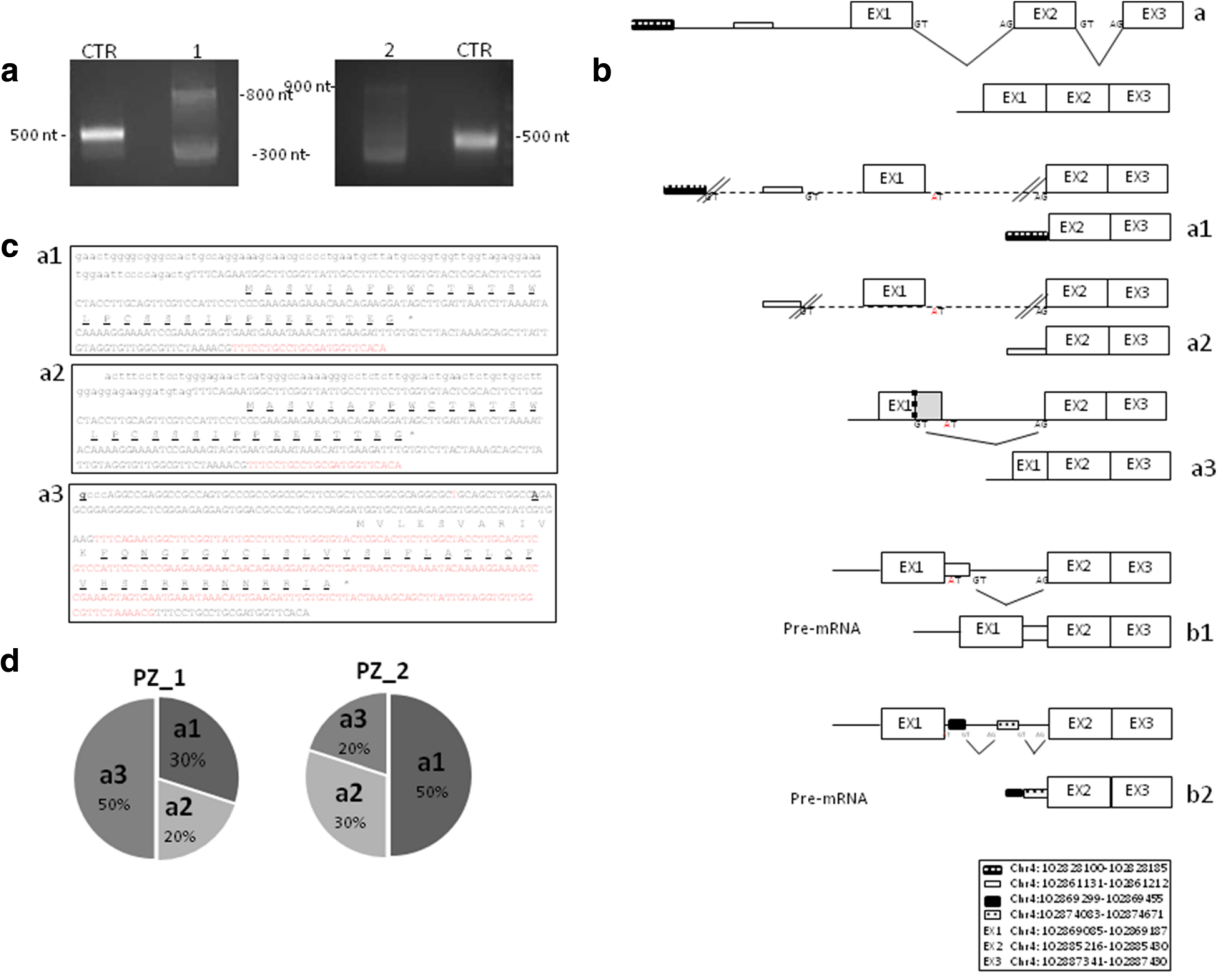
Multiple truncated and non functional CISD2 isoforms. **a** The 5′-RACE extended an expected 500 nt PCR product in the healthy control and two products of approximately 400 and 800–900 bases in the probands. **b** A schematic representation of the exon structure of wild-type (a) and mutant (a1-a3) *CISD2* variants, and putative pre-mRNAs (b1 and b2). The homozygous mutation in the donor splice of intron 1 generated multiple transcripts characterized by the whole or partial absence of exon 1 (a1-a3) and inefficiently spliced pre-mRNAs retaining a large segment of intron 1 (b1 and b2). **c** The sequencing resulting from the 400 nt 5′-RACE products. The cDNA and amino acids sequences are shown. The exons are indicated in uppercase letters and alternate colours. The 5′-UTR is indicated in *black* lowercase letters. a1 and a2: Short stretches of genomic DNA are joined to exon 2 perfectly spliced with exon 3. Genomic coordinates: Chr4:102,828,100–102,828,185 and Chr4:102,861,131–102,861,212, for a1 and a2 respectively. CpG island coordinates: Chr4:102,826,475–102,828,235. *Pugo* gene: Chr4:102,827,193–102,829,052. The ATG, predicted in the beginning of exon 2 (Chr4:102,885,223; NG_008636.2:21,246), shifted the open reading frame and introduced a downstream amino acid changes (underscored) in addition to premature stop. a3: The partial absence of exon 1 (Chr4:102,869,118–102,869,187; NG_008636.2:5141–5210) caused a frame shift, amino acids changes (underscored) and a premature stop. The transcriptional start sites are in bold letters and underscored, and they are located at 111 and 43 nt upstream the ATG for patient 1 and 2, respectively (position at 4997 and 5064 referred to NG_008636.2; position at 102868974 and 102,869,041 referred to Chr4). The SNP rs223332 (NG_008636.2: g.5052G > T) is indicated in *red* and lowercase letter. **d** The frequency of mutant isoforms in WFS2 patients

To validate the putative exon 1 absence demonstrated by RT-PCR, a 5′-RACE experiment was performed on total RNA derived from PBMCs of the patients and the healthy control. A gene-specific primer (GSP1) designed against exon 3 and a forward universal primer (UPM) were used for the PCR step that followed the cDNA synthesis in the 5′-RACE process. The expected PCR product of approximately 500 nt was extended in the healthy control, while smeared signals were detected in both homozygous samples, reinforcing the very low amount of mutant mRNA (data not shown). To increase specificity and sensitivity, a nested PCR with an inner GSP2 primer was performed on the primary PCR products which resulted in the extension of two fragments in both patients: one of approximately 400 nt in size, which was the more represented fragment, and one of 800–900 nt, which was less enriched (Fig. 2a).

The PCR products were cloned, and several independent clones were screened based on their molecular length. A direct sequencing analysis performed on ten different clones for each patient showed that the 400-nt product contained three different transcripts resulting from the whole or partial absence of exon 1 from the mature transcript. One product consisted of exon 2 perfectly spliced with exon 3 joined to a short stretch of genomic DNA sequence (86 nucleotides in size) located 57,022 nt upstream of the beginning of exon 2 (Fig. 2b, a1). Computer analysis indicated that this small segment of genomic DNA (Chr4:102,828,100–102,828,185, coordinates referred to GRCh38/hg38 of the human genome assembly, www.genome.ucsc.edu) was located within an CpG island (CpG:141, chr4:102,826,475 102,828,235) and partially overlapped with the *pugo* gene (Chr4:102,827,193–102,829,052) (Additional file 1: Figure S1). There is no NCBI RefSeq model for the *pugo* gene, but *Homo sapiens* cDNA sequences in GenBank that were aligned on the genome and clustered in a minimal non-redundant way by the manually supervised AceView program (www.ncbi.nlm.nih.gov/ieb/research/acembly), support two unspliced and three alternatively spliced variants. Among these variants, the unspliced *pugo* variant a, which is the only variant that putatively encodes a protein (DA090506 and DB032576), matched 86 nt of its non-coding region with the *CISD2* variant a1.

The second transcript, where exon 2 correctly spliced with exon 3, was joined to a short stretch of genomic DNA sequence (83 nucleotides in size) located at 24004 nucleotides upstream the beginning of exon 2 (Fig. 2b, a2). Computer analysis performed on this small segment of genomic DNA (Chr4:102,861,131–102,861,212) did not identify the existence of any genes. In both variants, in silico analysis (NetStart1, http://www.cbs.dtu.dk) predicted a translational start site in the beginning of exon 2 which resulted in a frame shift, introducing amino acids changes and a premature stop codon (Fig. 2c, a1 and a2). Finally, the third 5′-RACE product resulted in the exclusion of the last 70 bases of exon 1 from the mature transcript (Chr4:102,869,118–102,869,187; NG_008636.2:5141–5210), which shifted the open reading frame and introduced downstream amino acid changes in addition to a premature stop (Fig. 2b, a3 and c, a3). In this isoform, the transcriptional start site was mapped at 111 and 43 nt upstream of the ATG for patients 1 and 2 respectively (Fig. 2c, a3). The frequency of the mutant isoforms in each patient is reported in Fig. 2d. The coexistence of multiple mutant *CISD2* variants that diverged from one another by only the N-terminal region is also demonstrated by the sequence chromatograms from the 5′-RACE products that were not subcloned: the region spanning from exon 2 to exon 3 is represented by a single peak pattern, while the remaining N-terminal region is made up of multiple peak patterns (Additional file 2: Figure S2). The risk of false positive splice variant identification is very low since the three isoforms were detected in both siblings (two independent samples) although at different frequency.

The direct sequencing analysis performed on five clones for each patient showed that the upper 5′-RACE products may result in an inefficiently spliced pre-mRNAs that retained a large segment of intron 1 in the mRNA. In patient 1, the putative pre-mRNA retained the first 268 nt of intron 1 (Chr4:102,869,299–102,869,455; NG_008636.2: 5322–5478) in the final transcript, and the transcriptional start site was mapped to the same position identified in the smaller variant a3 (Fig. 2b, b1 and Additional file 3: Figure S3, b1). In patient 2, 646 nt region of intron 1 (Chr4102874083–102,874,671 and NG_008636.2: 10,204–10,694) was joined to exon 2 that was perfectly spliced with exon 3 (Fig. 2b, b2 and Additional file 3: Figure S3, b2). This product might be a prematurely terminated 5′-RACE fragment that did not reach the 5′ end of the template.

Together, the data demonstrated that the c.103 + 1G > A mutation functionally impaired mRNA splicing, producing multiple splice variants devoid of all or part of exon 1 that encoded for truncated non-functional isoforms.

### CISD2 protein expression

**Fig. 3:**
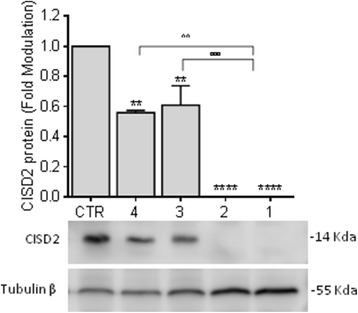
Analysis of *CISD2* protein expression in PBMCs. The CISD2 protein levels were analysed by Western blot analysis using polyclonal antibody raised against the CISD2 C-terminus encompassing the CDGSH iron sulfur domain. The CISD2 protein decreased approximately 50% in heterozygous samples compared with healthy PBMCs, and the signal was undetectable in the patient samples. β tubulin was used as loading control. The panels show representative images of three independent experiments. The histogram shows values normalized relative to a housekeeping protein and expressed as fold modulation compared with the healthy control. The error bars represent the mean ± SEM for three experiments. Ordinary one-way ANOVA, ****P < 0,0001

To further investigate the consequences of the c.103 + 1G > A mutation on the protein product, Western blot analysis was performed on PBMCs using a polyclonal antibody raised against the CISD2 C-terminus which encompasses the CDGSH iron sulfur domain absent in the mutant isoforms. The Western blot analysis did not reveal the presence of the protein in the patients, whereas a decrease of approximately 50% was observed in the heterozygous parents compared with that observed in the healthy control (Fig. 3).

Together, these data indicate that the c.103 + 1G > A mutation caused a complete loss of CISD2 protein expression in the WFS2 patients and a decrease of approximately 50% in the unaffected parents.

### Identification of SNP rs223332 in a WFS2 family

**Fig. 4:**
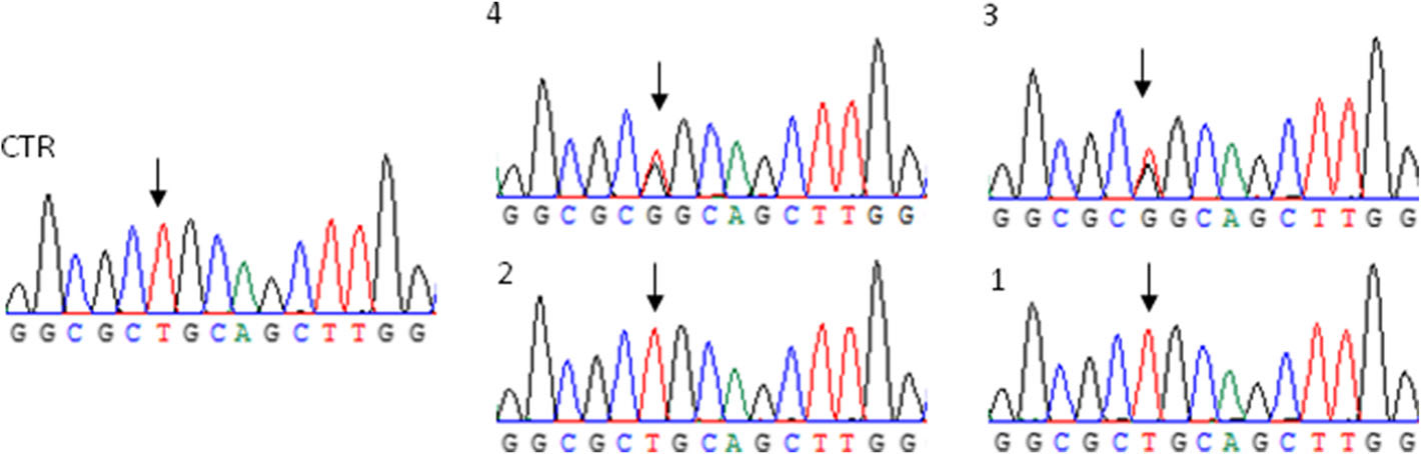
SNP genotyping. The sequence chromatograms comparing the probands (1 and 2) and the healthy control (CTR) that are homozygous for rs223332 and the parents (3 and 4) that are heterozygous for rs223332. The *black arrows* indicates the heterozygous and homozygous SNP

The direct sequencing performed on isoform a3 and putative pre-mRNA b1 showed that patient 1 was a homozygous carrier of single nucleotide polymorphism (SNP) rs223332 (Fig. 2c, a3; Additional file 2: Figure S2, b1). This SNP, which is a substitution of a guanine to thymine 56 nt upstream of the *CISD2* ATG (NG_008636.2:g.5052G > T), is annotated in the dbSNP database (http://www.ncbi.nlm.nih.gov/SNP/) with a minor allele frequency (G) of 40%. The SNP genotyping performed on the genomic DNA isolated from buccal swabs from all family members and a healthy control indicated that the parents were heterozygous for this SNP, while both patients and the healthy control were homozygous carriers (Fig. 4). Considering the high frequency in the population, this SNP alone might be inert, but it may exert a functional effect when associated with *CISD2* mutations or other simple and/or complex traits.

## Discussion

In this report, we demonstrate that the c.103 + 1G > A mutation, mapping in the donor site of the first intron of the *CISD2* gene and carried in two WFS2 patients, caused a complete loss of functional CISD2 protein. The RT-PCR coupled with 5′-RACE analyses demonstrated that the c.103 + 1G > A mutation functionally impaired the mRNA processing by generating multiple aberrant splice variants missing all or part of exon 1. The loss caused a shift in the open reading frame, introducing amino acids changes and a premature stop codon that resulted in multiple non-functional isoforms devoid of the transmembrane and the CDGSH domains. The lack of a functional CDGSH domain was also confirmed by Western blot analysis performed with a specific antibody directed towards this functional region.

Interestingly, intron 1 is extremely long, i.e. approximately 16 Kb in size and distributed over 23.82 Kb of genomic DNA. The c.103 + 1G > A mutation disrupts the splice site *consensus* sequence, the canonical dinucleotides GT that, together with multiple splicing factors, are important determinants for driving exon recognition, resulting in the inability of splicing factors to detect exon 1 and in the shift in the splice site recognition at 70, 7976 and 41,003 nucleotides upstream of the mutation. The resulting aberrant variants containing a frame shift and a premature termination are potential substrates for nonsense-mediated mRNA decay (NMD), a cellular quality control pathway that promotes the degradation of non-functional and deleterious transcripts [13]. Indeed, as based on the “50–55 nucleotide boundary rule” [14], a termination stop codons located more than 50 nucleotides upstream of the exon 2-exon 3 junction in the *CISD2* variants are predicted to activate NMD. This mRNA surveillance pathway may account for the drastic depletion in *CISD2* mRNA observed in patients and for the decreased of approximately 65% detected in the parents, which may explain the high instability of mutant mRNA. In addition, NMD may prevent the translation of the resulting encoded polypeptides that retained large-scale changes in amino acid sequences, premature stop codons and a loss of the functional domain. It is not excluded that other splice variants, beyond that identified by the 5′-RACE, might exist and that they could only be detected by more efficient and deep genome-wide investigations based on unbiased methods (i.e RNAseq).

Moreover, it is not excluded that the pathogenic splice variants detected by 5’RACE might be cell type specific, therefore it is important to extend this molecular approach to some of the other relevant cell types that are most linked to WFS2 (i.e pancreatic beta cells or neurons). The establishment of patient-derived induced pluripotent stem cells (iPSC) which can differentiate into neurons and pancreatic beta cells will represent a suitable disease model for investigating the c.103 + 1G > A mutation as well as for discovering novel therapeutic targets.

The complete loss of the CISD2 protein observed in patients closely resembles the molecular conditions of the Cisd2 knock out mouse providing further evidences that it could be a suitable animal model for the mechanistic investigation of the pathophysiological mechanism of WFS2. Notably, the four cases of WFS2 with mutations in the *CISD2* gene resulted in different aberrant proteins that share the loss of the evolutionary conserved CDGSH domain, underscoring the crucial importance of this functional domain to maintain and preserve the mitochondrial homeostasis of the cells, particularly of the pancreatic beta cells and neurons, which are the main affected cells in WFS2.

## Conclusions

In summary, our study demonstrated that the c.103 + 1G > A mutation, mapping in the donor site of the first *CISD2* intron of two WFS2 patients, functionally impaired mRNA splicing and produced multiple splice variants characterized by the complete or partial loss of exon 1. These losses produced an aberrant transcripts harbouring a premature stop codon that renders them a potential substrates for NMD. The affected variants resulted in the loss of functional CISD2 protein.

## Additional files

Additional file 1: Figure S1. Schematic representation of the overlapping region between the *CISD2* variant a1, the CpG island and the *pugo* gene: *CISD2* variant a1: Chr4:102,828,100–102,828,185, CpG island: CpG: 141, Chr4:102,826,475–102,828,235 and the *pugo* gene: Chr4:102,827,193–102,829,052. (TIFF 61 kb)

Additional file 2: Figure S2. Chromatograms of the 400 nt-5′-RACE products not subjected to subcloning. (TIFF 155 kb)

Additional file 3: Figure S3. The sequencing resulting from the 800 and 900 nt 5′-RACE products. The cDNA and amino acids sequences are shown. Exons are indicated in uppercase letters and alternate colours. The 5′-UTR and partial region of intron 1 are indicated in black lowercase letters. b1: The putative pre-mRNA retained 286 nt of intron 1 (Chr4:102,869,299–102,869,455; NG_008636.2: 5322–5478) in the final transcript. The transcriptional start site is in bold letters (position at 4997 referred to NG_008636.2 and position at 102868974 referred to Chr4). The SNP rs223332 (NG_008636.2: g.5052G > T) is indicated in red. The homozygous mutation (NM_001008388.4:c.103 + 1G > A) is in bold letters and underlined. b2: The putative pre-mRNA retained 646 nt of intron 1 (Chr4102874083–102,874,671; NG_008636.2: 10,204–10,694) in the final transcript. (TIFF 68 kb)

## Abbreviations

CISD2: CDGSH iron sulfur domain 2
ER: Endoplasmic reticulum
MAMs: Mitochondria-associated ER membranes
MOM: Mitochondrial outer membrane
NMD: Nonsense mRNA decay
PBMCs: Peripheral blood mononuclear cells
ROS: Reactive oxygen species
WFS2: Wolfram syndrome type 2

## References

[1] Amr S, Heisey C, Zhang M, Xia XJ, Shows KH, Ajlouni K (2007). A homozygous mutation in a novel zinc-finger protein, ERIS, is responsible for Wolfram syndrome 2. Am J Hum Genet.

[2] Mozzillo E, Delvecchio M, Carella M, Grandone E, Palumbo P, Salina A (2014). A novel CISD2 intragenic deletion, optic neuropathy and platelet aggregation defect in Wolfram syndrome type 2. BMC Med Genet.

[3] Rondinelli M, Novara F, Calcaterra V, Zuffardi O, Genovese S (2015). Wolfram syndrome 2: a novel *CISD2* mutation identified in Italian siblings. Acta Diabetol.

[4] Puca AA, Daly MJ, Brewster SJ, Matise TC, Barrett J, Shea-Drinkwater M (2001). A genome-wide scan for linkage to human exceptional longevity identifies a locus on chromosome 4. Proc Natl Acad Sci U S A.

[5] Wiley SE, Paddock ML, Abresch EC, Gross L, van der Geer P, Nechushtai R (2007). The outer mitochondrial membrane protein mitoNEET contains a novel redox-active 2Fe-2S cluster. J Biol Chem.

[6] Wang CH, Kao CH, Chen YF, Wei YH, Tsai TF (2014). Cisd2 mediates lifespan: is there an interconnection among Ca^2^? Homeostasis, autophagy, and lifespan?. Free Radic Res.

[7] Tamir S, Paddock ML, Darash-Yahana-Baram M, Holt SH, Sohn YS, Agranat L (1853). Structure-function analysis of NEET proteins uncovers their role as key regulators of iron and ROS homeostasis in health and disease. Biochim Biophys Acta.

[8] Sohn YS, Tamir S, Song L, Michaeli D, Matouk I, Conlan AR (2013). NAF-1 and mitoNEET are central to human breast cancer proliferation by maintaining mitochondrial homeostasis and promoting tumor growth. Proc Natl Acad Sci U S A.

[9] Holt SH, Darash-Yahana M, Sohn YS, Song L, Karmi O, Tamir S (2016). Activation of apoptosis in NAF-1-deficient human epithelial breast cancer cells. J Cell Sci.

[10] Danielpur L, Sohn YS, Karmi O, Fogel C, Zinger A, Abu-Libdeh A (2016). GLP-1-RA corrects mitochondrial labile iron accumulation and improves β-cell function in type 2 Wolfram syndrome. J Clin Endocrinol Metab.

[11] Chen YF, Kao CH, Kirby R, Tsai TF (2009). Cisd2 mediates mitochondrial integrity and life span in mammals. Autophagy.

[12] Rouzier C, Moore D, Delorme C, Lacas-Gervais S, Ait-El-Mkadem S, Fragaki K (2017). A novel *CISD2* mutation associated with a classical Wolfram syndrome phenotype alters Ca2+ homeostasis and ER-mitochondria interactions. Hum Mol Genet.

[13] Kervestin S, Jacobson A (2012). NMD: a multifaceted response to premature translational termination. Nat Rev Mol Cell Biol.

[14] Hwang J, Kim YK (2013). When a ribosome encounters a premature termination codon. BMB Rep.

