# Supplementary figures and images for: A donor splice site mutation in CISD2 generates multiple truncated, non-functional isoforms in Wolfram syndrome type 2 patients
Source: BMC Med Genet. 2017 Dec 13;18:147. doi: 10.1186/s12881-017-0508-2 (PMC5729406; doi:10.1186/s12881-017-0508-2)

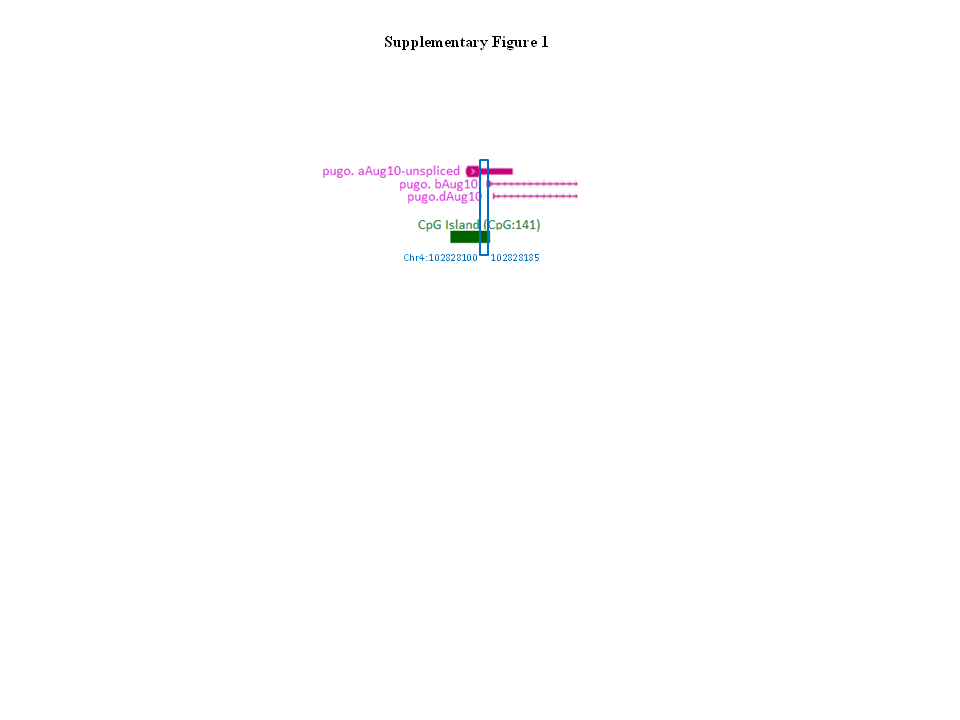

Supplement: Supplementary file 1 — Schematic representation of the overlapping region between the CISD2 variant a1, the CpG island and the pugo gene: CISD2 variant a1: Chr4:102,828,100–102,828,185, CpG island: CpG: 141, Chr4:102,826,475–102,828,235 and the pugo gene: Chr4:102,827,193–102,829,052. (TIFF 61 kb) [file 12881_2017_508_MOESM1_ESM.tif]

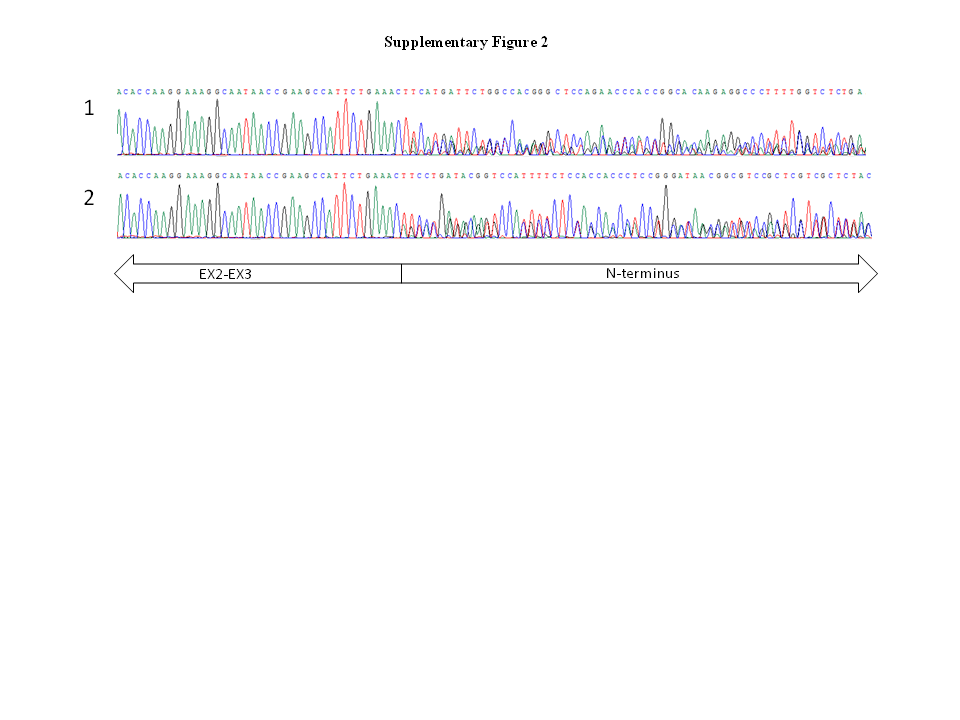

Supplement: Supplementary file 2 — Chromatograms of the 400 nt-5′-RACE products not subjected to subcloning. (TIFF 155 kb) [file 12881_2017_508_MOESM2_ESM.tif]

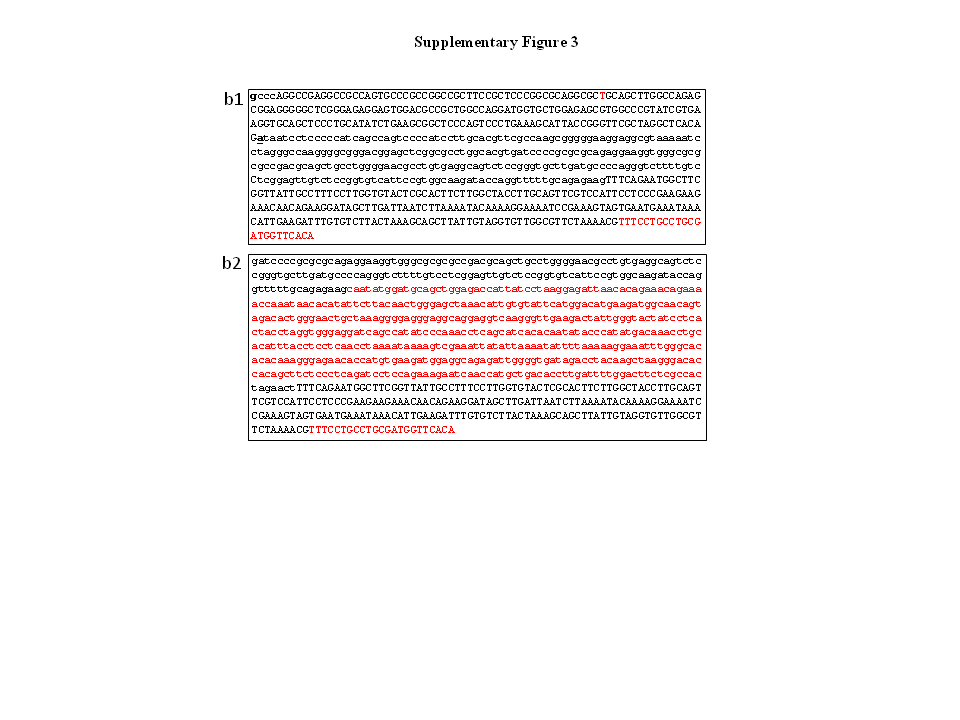

Supplement: Supplementary file 3 — The sequencing resulting from the 800 and 900 nt 5′-RACE products. The cDNA and amino acids sequences are shown. Exons are indicated in uppercase letters and alternate colours. The 5′-UTR and partial region of intron 1 are indicated in black lowercase letters. b1: The putative pre-mRNA retained 286 nt of intron 1 (Chr4:102,869,299–102,869,455; NG_008636.2: 5322–5478) in the final transcript. The transcriptional start site is in bold letters (position at 4997 referred to NG_008636.2 and position at 102868974 referred to Chr4). The SNP rs223332 (NG_008636.2: g.5052G > T) is indicated in red. The homozygous mutation (NM_001008388.4:c.103 + 1G > A) is in bold letters and underlined. b2: The putative pre-mRNA retained 646 nt of intron 1 (Chr4102874083–102,874,671; NG_008636.2: 10,204–10,694) in the final transcript. (TIFF 68 kb) [file 12881_2017_508_MOESM3_ESM.tif]
